# Experimental Design and Optimization of Recovering Bioactive Compounds from *Chlorella vulgaris* through Conventional Extraction

**DOI:** 10.3390/molecules27010029

**Published:** 2021-12-22

**Authors:** Ioulia Georgiopoulou, Soultana Tzima, Georgia D. Pappa, Vasiliki Louli, Epaminondas Voutsas, Kostis Magoulas

**Affiliations:** Laboratory of Thermodynamics and Transport Phenomena, School of Chemical Engineering, National Technical University of Athens (NTUA), Zografou Campus, 15780 Athens, Greece; tani-tzim@hotmail.com (S.T.); gepappa@central.ntua.gr (G.D.P.); svlouli@chemeng.ntua.gr (V.L.); evoutsas@chemeng.ntua.gr (E.V.); mag@chemeng.ntua.gr (K.M.)

**Keywords:** microalgae, green solvents, carotenoids, chlorophylls, phenolics, antioxidant activity, extraction optimization

## Abstract

Microalgae contain an abundance of valuable bioactive compounds such as chlorophylls, carotenoids, and phenolics and, consequently, present great commercial interest. The aim of this work is the study and optimization of recovering the aforementioned components from the microalgae species *Chlorella vulgaris* through conventional extraction in a laboratory-scale apparatus using a “green” mixture of ethanol/water 90/10 *v*/*v*. The effect of three operational conditions—namely, temperature (30–60 °C), duration (6–24 h) and solvent-to-biomass ratio (20–90 mL_solv_/g_biom_), was examined regarding the extracts’ yield (gravimetrically), antioxidant activity, phenolic, chlorophyll, and carotenoid contents (spectrophotometric assays), as well as concentration in key carotenoids, i.e., astaxanthin, lutein, and β-carotene (reversed-phase–high-performance liquid chromatography (RP–HPLC)). For this purpose, a face-centered central composite design (FC-CCD) was employed. Data analysis resulted in the optimal extraction conditions of 30 °C, for 24 h with 37 mL_solv_/g_biom_ and validation of the predicted models led to 15.39% *w*/*w* yield, 52.58 mg_extr_/mg_DPPH_ (IC50) antioxidant activity, total phenolic, chlorophyll, and carotenoid content of 18.23, 53.47 and 9.92 mg/g_extr_, respectively, and the total sum of key carotenoids equal to 4.12 mg/g_extr_. The experimental data and predicted results were considered comparable, and consequently, the corresponding regression models were sufficiently reliable for prediction.

## 1. Introduction

Microalgae have gained considerable scientific and commercial interest in the fields of energy, food, nutraceuticals, pharmaceuticals, and cosmetics over the last few decades [1,2,3]. Initially, studies were focused on the recovery of their lipid-rich extracts and utilization in biofuel production. However, attention has now been drawn to the utilization of their high-demand natural compounds, such as polyunsaturated fatty acids, pigments, phenolic compounds, vitamins, sterols, proteins, and enzymes [2,4].

One of the most dominant microalgae species in terms of production rate is Chlorella, ranked second right after *Arthrospira* with annual global production of 5.000 against 12,000 tn. [3] *Chlorella* genus, and specifically *Chlorella vulgaris* (*C. vulgaris*) species, was first discovered by M.W. Beijerinck in 1890, but the starting point of industrial production was after 1960 in Japan [5,6]. Today *C. vulgaris*, along with *C. pyrenoidosa* and *C. luteoviridis*, are enlisted in the EU Novel Food Catalog [7], and *Chlorella* is commercially used as a food additive, supplement, pigment, food emulsion, and animal feed [6,8].

*C. vulgaris* is a high-potential source of bioactive substances such as pigments (chlorophyll and carotenoids), phenolic compounds, etc. Chlorophyll is the most abundant pigment found in the *C. vulgaris* cells and is considered superior to synthetic dyes, as it is a natural pigment suitable for food and cosmetic use [6]. Chlorophyll also presents healing effects suitable for ulcer treatment and liver recovery and contributes to accelerated cell growth and repair [9,10]. Carotenoids, which exhibit significant antioxidant activity [11] and contribute to strengthening the immune system [12], are also produced from microalgae [13]. Carotenoids that commonly occur in *C. vulgaris* biomass are β-carotene, a precursor of vitamin A [14], lutein, astaxanthin, canthaxanthin, and violaxanthin, ingredients with stimulating, antioxidant and anticancer action [15,16]. Phenolic compounds are present in microalgae cells and are known for their antioxidant, antifungal, and antibacterial activity. It has also been found that phenolics in *C. vulgaris* formulations contribute to their antidiabetic effect [17,18].

A simple and widely known separation method is the conventional solid–liquid extraction (SLE). According to the process mechanisms, when SLE is performed the following stages can occur: solvent transfer from the bulk solution to matrix surface, solvent penetration into the matrix, dissolution of solutes into the solvent, solute carriage to matrix surface, and diffusion of solutes into the bulk solution. Moreover, factors such as temperature, solvent selection, solid-to-liquid ratio, and time for sufficient contact of solvent and matrix are considered important factors that affect SLE efficiency [19].

Substances such as chlorophylls, carotenoids, and phenolic compounds have been detected individually in *C. vulgaris* extracts obtained by conventional solid–liquid extraction methods with organic solvents. For example, Mendes et al. [20,21] recovered 0.03 and 0.04% *w*/*w* carotenoids from *C. vulgaris* via conventional extraction with n-hexane and acetone, respectively. Gouveia et al. [22] and Palavra et al. [23] determined total carotenoid content, 0.426 and 0.43%, respectively, after exhaustive extraction of *C. vulgaris* biomass with acetone. Li et al. [24] also performed exhaustive lutein extraction from *C. vulgaris* with dichloromethane and achieved 91% recovery. The crude extract contained ~30% lutein, while further purification of lutein led up to 90% purity. Moreover, Kitada et al. [25] applied Soxhlet extraction with ethanol to *C. vulgaris* biomass and recovered ~2 mg/g_sample_ lutein and ~18 mg/g_sample_ chlorophylls, while Ruen-ngam [26], determined the total lutein content of *C. vulgaris* to be equal to 7.9 ± 0.54 mg/g_biom_ using chloroform. Cha et al. [27] studied the conventional extraction of *C. vulgaris* biomass using acetone, hexane, and ethanol 50–100% and concluded that the ethanol/water 90:10 *v*/*v* mixture was the best solvent for carotenoid and chlorophyll extraction. Specifically, conventional extraction of *C. vulgaris* with 90% *v*/*v* ethanol at ambient temperature, in the absence of light and for a period of 6 h resulted in the highest extraction yield (~30%) and the extract contained 2.97 ± 0.31 mg/g_sample_ lutein, 0.08 ± 0.01 mg/g_sample_ β-carotene, 4.26 ± 0.53 mg/g_sample_ chlorophyll α and 2.58 ± 0.09 mg/g_sample_ chlorophyll b. Furthermore, the same method was applied later by Cha et al. [28] and led to a 25% yield, and the extract had a total phenolic content of ~7 mg_GA_/g_extr_ and an equivalent antioxidant capacity of 90 μmol_Trolox_/g_extr_.

During algae-based extraction of bioactive compounds, toxic and hazardous organic solvents (e.g., acetone, methanol, diethyl ether, chloroform, hexane) are commonly used [29]. However, the necessity to develop sustainable processes, especially in the fields of food, nutraceuticals, pharmaceuticals, and cosmetics, requires compliance with the concept of “green” chemistry and “green” extraction. The main objective of “green” chemistry and basic principles of “green” extraction include the selection of innovative and renewable raw materials and alternative environmentally friendly solvents, reduction in energy consumption, application of safe and robust processes, recovery of pure denatured and biodegradable extracts and by-product utilization [30,31].

Therefore, solvent selection coupled with proper processes in terms of “green” chemistry contributes to a promising holistic approach for the extraction of algal bioactive compounds. “Green” solvents comprise classical solvents such as bio-based water and ethanol and the renewably sourced 1-butanol and ethyl acetate, as well as novel solvents including supercritical fluids, ionic liquids, and natural deep eutectic solvents [32,33]. Water and ethanol are highly recommended “green” solvents, preferred in terms of ensuring health and safety, and environmental protection [34]. Moreover, according to Cha et al. [27,28], aq. ethanol 90% *v*/*v* already showed an appreciable advantage in terms of *C. vulgaris* bioactive compound recovery over other ratios as well as different organic solvents. In conclusion, the solvents of ethanol and water, the microalgae biomass of *C. vulgaris*, and the simple conventional extraction method are certainly considered suitable according to the principles of “green” extraction [30].

The novelty of this work lies in the comprehensive, simultaneous study of the effect of three process parameters on six different features of *C. vulgaris* extracts that eventually allows response prediction under acceptable confidence levels. The proposed method includes multiple bioactive compound recovery from the microalgae species *C. vulgaris* through conventional extraction in a laboratory-scale apparatus using a solvent mixture of ethanol/water 90/10 *v*/*v*. The effect of extraction temperature (30–60 °C), duration (6–24 h), and solvent-to-biomass ratio (20–90 mL_solv_/g_biom_) was examined according to proper experimental design. The development of the experimental design, the analysis of variance (ANOVA) of all the examined responses, and the optimization of the proposed extraction method were based on the advantageous and widely applied response surface methodology (RSM) of face-centered central composite design (FC-CCD). FC-CCD is considered a useful tool for building a model without the need for a full-factorial design [35]. All extracts were evaluated in terms of their yield (gravimetrically), antioxidant activity, total phenolic, chlorophyll, and carotenoid content using spectrophotometric assays and content of selected carotenoids, i.e., astaxanthin, lutein, and β-carotene using reversed-phase–high-performance liquid chromatography (RP–HPLC). Finally, ANOVA models’ verification of all examined responses was performed in order to ensure that the generated equations were suitable for the prediction of the extract’s composition in bioactive compounds and antioxidant activity.

## 2. Results and Discussion

### 2.1. Biomass Profile

According to Table 1, chemical analysis of the examined biomass of *C. vulgaris* showed that the main components were proteins, followed by carbohydrate and lipid compounds. Protein, lipid, and carbohydrate contents of *C. vulgaris* are highly dependent on the applied growth conditions and represent 42–58, 5–40, and –55% dw, respectively [6,36,37]. Therefore, the resulting composition is consistent with the literature. Moreover, ash content, an indication of the inorganic matter, i.e., minerals, was comparable to the ash content of *C. vulgaris* found in the literature, ranging from 6.3 to 15.8% dw [36,37,38]. Moisture level did not exceed 15%, ensuring anaerobic microbial activity inhibition [39] and allowing safe storage of the biomass for as long as the experiments were performed.

### 2.2. Extraction and Recovery of Bioactive Compounds

A total of 18 extraction experiments were performed, and the results are presented in Table 2. The extraction yield ranged from 11.06 to 21.01 ± 0.54% *w*/*w*. The transition of the examined variables from low (30 °C, 6 h, 20 mL_solv_/g_biom_) to high levels (60 °C, 24 h, 90 mL_solv_/g_biom_) almost doubled the value. The antioxidant activity of the *C. vulgaris* extracts varied from 34.73 to 79.41 ± 5.52 mg_extr_/mg_DPPH_. Extract obtained under the low levels of extraction’s temperature and duration (30 °C, 6 h, 90 mL_solv_/g_biom_) showed the highest antioxidant activity, while the lowest, more than half, occurred by applying the high levels of the corresponding parameters (60 °C, 24 h, 20 mL_solv_/g_biom_).

Moreover, total phenolic content received values between 7.07 and 27.35 ± 3.61 mg_GA_/g_extr_. Temperature decrease from 60 to 30 °C while maintaining high levels of extraction duration and solvent-to-biomass ratio (24 h, 90 mL_solv_/g_biom_) increased phenolic content almost four and a half times. Regarding the total chlorophyll content of the extracts, it ranged from 27.86 to 50.43 ± 2.48 mg/g_extr_. Both minimum and maximum values occurred at low levels of extraction temperature and duration (30 °C, 6 h), revealing the positive effect of decreasing the solvent-to-biomass ratio from 90 to 20 mL_solv_/g_biom_, which doubled the chlorophyll content.

Finally, selected–astaxanthin, lutein, and β-carotene–and total carotenoid content ranged from 1.88 to 4.87 ± 0.13 mg/g_extr_ and 6.36 to 9.90 ± 0.52 mg/g_extr_, respectively. The low level of extraction temperature and solvent-to-biomass ratio (30 °C, 20 mL_solv_/g_biom_) and high level of duration (24 h) led to the extract with both higher selected and total carotenoid content. The increase in extraction temperature and solvent-to-biomass ratio to the high examined levels (60 °C, 90 mL_solv_/g_biom_) caused a significant decrease in selected carotenoid content, almost two and a half times. However, the complete inversion of all examined parameters (60 °C, 6 h, 90 mL_solv_/g_biom_) led to a decrease in total carotenoid content, almost one and a half times.

**Figure 1:**
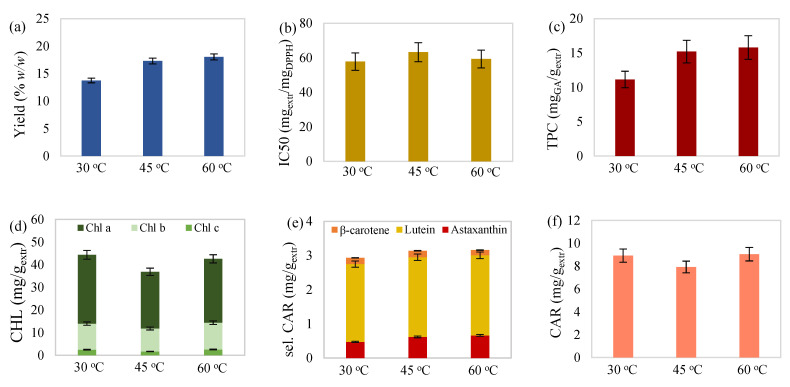
(**a**) Yield, (**b**) antioxidant activity, (**c**) total phenolic content, (**d**) total chlorophylls, (**e**) selected carotenoids (astaxanthin, lutein, and β-carotene), and (**f**) total carotenoid content of maceration extract from *C. vulgaris* as a function of the extraction temperature for 15 h with 55 mL/g solvent-to-biomass ratio.

**Figure 2:**
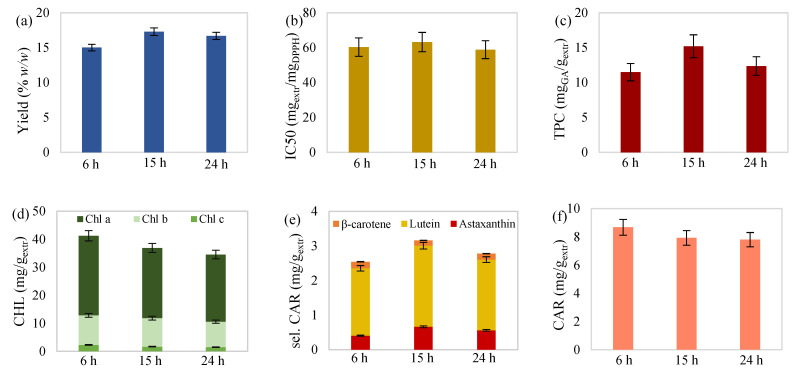
(**a**) Yield, (**b**) antioxidant activity, (**c**) total phenolic content, (**d**) total chlorophylls, (**e**) selected carotenoids (astaxanthin, lutein, and β-carotene), and (**f**) total carotenoid content of maceration extract from *C. vulgaris* as a function of the extraction duration at 45 °C with 55 mL/g solvent-to-biomass ratio.

**Figure 3:**
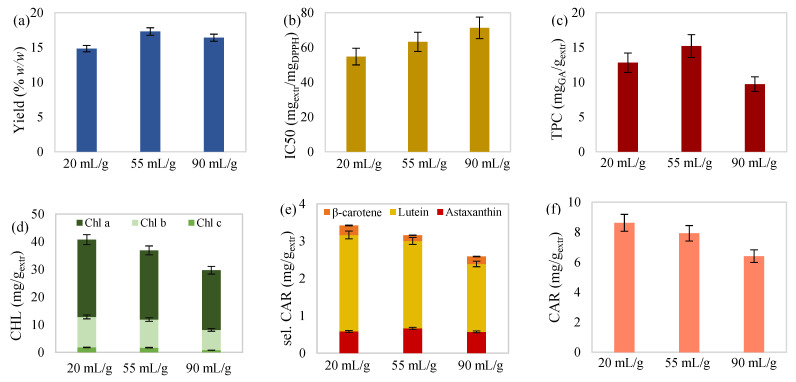
(**a**) Yield, (**b**) antioxidant activity, (**c**) total phenolic content, (**d**) total chlorophylls, (**e**) selected carotenoids (astaxanthin, lutein, and β-carotene), and (**f**) total carotenoid content of maceration extract from *C. vulgaris* as a function of solvent/biomass ratio at 45 °C for 15 h.

**Figure 4:**
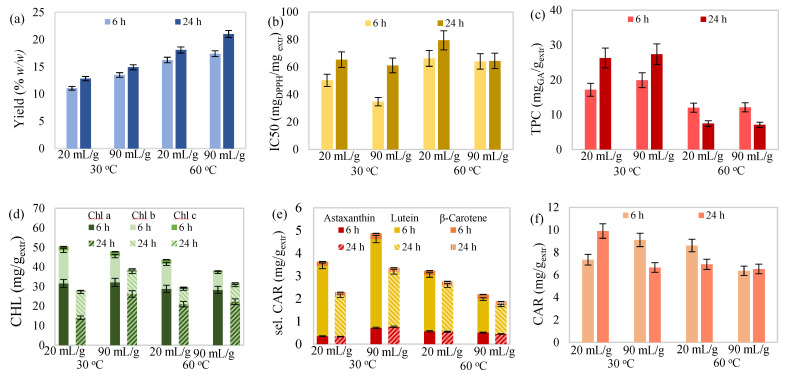
(**a**) Yield, (**b**) antioxidant activity, (**c**) total phenolic content, (**d**) total chlorophylls, (**e**) selected carotenoids (astaxanthin, lutein, and β-carotene) and (**f**) total carotenoid content of maceration extract from *C. vulgaris* as a function of extraction temperature, duration and solvent/biomass ratio.

Moreover, carotenoid determination of the extracts showed that lutein prevailed over the other two selected carotenoids (Figure 1e, Figure 2e, Figure 3e and Figure 4e). Percentages ranged from 66.86 to 85.10% for lutein, followed by astaxanthin, with 9.74–23.90%, and β-carotene, with 4.25–9.24%. The proportion of selected over total carotenoids ranged from 19.23 to 42.08% for lutein, followed by astaxanthin, with 3.65–11.44%, and β-carotene, with 1.72–3.11%. Likewise, referring to chlorophyll content, chlorophyll a was dominant (Figure 1d, Figure 2d, Figure 3d and Figure 4d), with percentages ranging from 50.49 to 74.43%, followed by chlorophyll b, with 23.45–41.22%, and chlorophyll c, with 2.12–6.05%.

In an attempt to compare experimental results with related literature, the work of Cha et al. [27,28], as mentioned earlier, was considered most relevant, as conventional extraction with aq. ethanol 90% *w*/*w* was carried out at ambient temperature for 6 h with a 100 mL/g solvent-to-biomass ratio. The most similar, in terms of extraction conditions, the experiment was the 6 h extraction at 30 °C with 90 mL_solv_/g_biom_ solvent-to-biomass (Table 2; Run 3). The slightly increased solvent-to-biomass ratio mentioned in the literature might be responsible for the elevated extraction yield noted since this offers a greater concentration gradient to the solvent/biomass system and, consequently, contributes to faster and more intense diffusion phenomena [40]. Regarding the rest of the compared responses, β-carotene, chlorophyll a, and chlorophyll b presented relatively low discrepancies. Slightly different extraction temperature (30 °C instead of ambient temperature) and solvent-to-biomass ratio (90 instead of 100 mL_solv_/g_biom_), as well as experimental errors, may justify such discrepancies. On the other hand, lutein and total phenolic content showed significant deviations (>40%) that can be attributed to different growth, harvest, drying, and storage conditions of the biomass.

#### 2.2.1. Effect of Temperature

Visualizing the results through Figure 1, Figure 2 and Figure 3 also led to some conclusions regarding the individual effect of the examined variables on all responses. According to Figure 1, temperature increase on 15 h extractions with 55 mL_solv_/g_biom_ solvent-to-biomass ratio caused an increase in extraction yield. In general, temperature rise contributes to the solvent’s viscosity reduction while increasing the solubility and diffusion coefficients of biomass compounds, and as a result, extraction yield is enhanced [41]. Temperature increase also led to a reasonable elevated phenolic content since higher temperatures benefit phenolic extraction [42].

Regarding the selected carotenoid content, temperature appeared to have a slightly positive effect. Reduced values of chlorophyll and total carotenoid content were observed as the temperature increased from 30 to 45 °C, while the transition to 60 °C led to subsequent pigment increase. Reduced total chlorophyll and carotenoid content at the intermediate temperature could also justify the behavior of antioxidant activity, which seemed to improve at the extreme temperatures examined. A similar reduction phenomenon of carotenoids and chlorophylls in the examined range of 30–47 °C has been noted in the study of Babadi et al. [43], where extraction of chlorophylls and carotenoids from *Chlorococcum humicula* with liquified dimethyl ether was evaluated. Reduced pigment content was considered to occur due to the breakdown of chlorophylls, such as pheophorbide formation from chlorophyll a [44], and degradation of any heat-sensitive carotenoids. However, the subsequent increase in chlorophylls resembles the behavior of chlorophyll content in the study of Kong et al. [10], where ultrasound-assisted extraction of *C. vulgaris* with an aqueous ethanolic solution at 60 °C enhanced chlorophyll extraction, as compared with 40 °C.

Additionally, aside from increased pigment content, the carotenoid isomerization phenomenon, which occurs in the case of heating, could also justify antioxidant activity improvement at elevated temperatures (60 °C). More specifically, heat treatment is known for promoting isomerization of the naturally all trans-carotenoids to cis-forms. According to Honda et al. [45], cis-isomerization may have an ambiguous effect on the antioxidant activity of carotenoids and, consequently, the extract’s quality. For example, depending on the assay method applied, cis-conversion of β-carotene was considered responsible for both improvement and deterioration of antioxidant activity [46,47]. Contrariwise, cis-isomers of lutein, astaxanthin, and canthaxanthin, predominate carotenoids in *C. vulgaris* present extracts, presented higher antioxidant activity than their trans-isomers [48,49,50].

#### 2.2.2. Effect of Time

As shown in Figure 2, the increase in the duration of extractions carried out at 45 °C with 55 mL_solv_/g_biom_ solvent-to-biomass ratio caused a slight yield increase. Longer contact time between solvent and biomass can enhance the mass transfer phenomena and extraction’s efficiency. Extract’s antioxidant activity did not appear to be significantly affected by extraction time variation, while its increase seemed to deteriorate chlorophylls and slightly reduce the carotenoid content. Although increased extraction time contributes to improved mass yield, pigment decrease could occur due to denaturing effects caused by the prolonged exposure to oxygen and elevated temperature [51].

On the other hand, phenolic content showed a maximum value in the intermediate extraction time of 15 h. A similar phenomenon has been observed by Casazza et al. [52], where ethanolic solid–liquid extraction of grape seeds with 0.3 g/mL solid/liquid ratio at room temperature led to an initial increase in total phenolics (gallic acid equivalent), from 9 to 19 h, followed by a decrease after the prolonged extraction time of 29 h. This behavior was justified due to the extended presence of oxygen that led to oxidative phenomena.

Selected carotenoid content also followed an upward and then downward trend. A relevant study of carotenoid extraction from tomato waste with various organic solvents, including ethanol, by Strati and Oreopoulou [41], concluded that carotenoids’ initial high extraction rate decreased with time until equilibrium was approached. The reduced extraction rate after 16 h extraction, in combination with long oxygen exposure, could be responsible for the cessation of carotenoid recovery as well as denaturation effects and, therefore, led to this fluctuation of selected carotenoids.

#### 2.2.3. Effect of Solvent-to-Biomass Ratio

Ratio increase, when examined on 15 h extractions at 45 °C, contributed to a slight improvement in extraction yield. The higher the solvent-to-biomass ratio, the greater the concentration gradient of the solvent/biomass system, which contributes to faster diffusion of the dissolved molecules outside the microalgae cells [40]. Nevertheless, increasing solvent-to-biomass ratio caused a total decrease in the rest examined responses, along with phenolic content, which, however, increased at the intermediate ratio value (Figure 3).

In the studies of Gui-you et al. [53] and Zhang et al. [54], the effect of solid/liquid ratio was studied on the content of bioactive compounds and antioxidant activity of extracts obtained from *Daldinia concentrica* and *Asparagus officialis*, respectively. Both studies concluded that the examined responses improved with ratio increase up to a certain value and then deteriorate when exceeding it. This downturn, also observed in the present study, could be due to extraction of various other components, not necessarily of the same potency, thereby reducing the concentration of the examined bioactive compounds and, consequently, extract’s antioxidant activity.

#### 2.2.4. Synergistic Effect of Temperature, Time, and Solvent-to-Biomass Ratio

The valuation of the combined effects of the extraction temperature, duration, and solvent-to-biomass ratio was attempted, which is shown in Figure 4. Extraction yield showed improvement proportional to all three independent variables. For the rest of the examined responses, there was no obvious effect regarding the synergy of temperature, duration, and ratio, except for some general conclusions. More specifically, a decrease in extraction temperature and duration seemed to favor antioxidant activity and total phenolic content, while a decrease in extraction temperature and ratio favored chlorophyll content. Finally, improvement of the selected carotenoid content was observed with simultaneous reduction in all independent variables. Since understanding the synergistic effects of the extraction temperature, duration, and solvent-to-biomass ratio is considered a complex process, the use of a proper model that fits well to the experimental data could facilitate the comprehension of their effect.

### 2.3. Optimization of the Extraction Process

#### 2.3.1. Regression Model Equation Fitting

In order to fit the experimental data of Table 2, Equations (9) and (10), presented in Section 3.3.2, were used. Responses were expressed with equations of 5 to 10 terms including the intercept, while the third-degree terms were used only where required. The addition of cubic terms, where deemed necessary, as well as the essential presence of factors required to support hierarchy, consequently increased the total number of model terms. Regression model equations are expressed in real terms. In the cases of using response transformation, the regression model equation of the transformation is followed by the actual response equation. The expressions of yield, Equation (1), antioxidant activity, Equations (2) and (3), total phenolic content, Equation (4), total chlorophyl content, Equations (5) and (6), selected carotenoid contents, Equation (7) and total carotenoid content (8) are presented below.

Yield = 3.4672 + 0.1650 × T + 0.1145 × t + 0.1154 × ratio − 7.8423 × 10^−4^ × ratio^2^ (1)

IC50′ = 3.8629 − 0.0107 × T + 0.1640 × t − 0.0541 × ratio − 6.7583 × 10^−3^ × T × t + 2.5282 × 10^−3^ × T × ratio + 2.7491 × 10^−4^ × T^2^ + 6.8524 × 10^−5^ × T^2^ × t − 2.7579 × 10^−5^ × T^2^ × ratio (2)

IC50 = e^IC50′^ (3)

TPC = 66.9280 − 1.1168 × T − 9.2051 × t + 1.2511 × 10^−3^ × ratio + 0.1939 × T × t + 0.3461 × t^2^ − 7.2698 × 10^−3^ × T × t^2^ (4)

CHL’ = 3.2559 − 0.0307 × T − 0.1071 × t + 9.7216 × 10^−4^ × ratio − 5.4560 × 10^−3^ × T × t + 2.3853 × 10^−4^ × t × ratio − 3.2808 × 10^−4^ × T^2^ − 8.4979 × 10^−5^ × ratio^2^ + 5.6995 × 10^−5^ × T^2^
× t(5)

CHL = e^CHL’^ (6)

sel. CAR = 6.7000 − 0.0754 × T − 0.4492 × t − 0.0351 × ratio + 0.0113 × T × t + 4.9602 × 10^−4^ × T × ratio + 0.0211 × t^2^ − 5.0663 × 10^−4^ × T × t^2^ (7)

CAR = 4.0345 − 0.0614 × T + 0.5595 × t + 0.2183 × ratio − 0.0115 × T × t − 2.9940 × 10^−3^
× T × ratio − 9.4095 × 10^−3^ × t × ratio + 2.4303 × 10^−3^ × T^2^ − 7.5348 × 10^−4^ × ratio^2^ + 1.8110 × 10^−4^ × T × t × ratio (8)

where yield is expressed in % *w*/*w* (g_extr_/g_biom_), antioxidant activity in m_gextr_/mg_DPPH_, total phenolic content in mg_GA_/g_extr_, and total chlorophyll, carotenoid, and selected carotenoid content in mg/g_extr_, while T, t, and ratio stand for the extraction temperature (°C), duration (h), and solvent-to-biomass ratio (mL_solv_/g_biom_).

In conclusion, according to the ANOVA results (Table A1, Appendix A) and regarding individual impact study, temperature variation caused the greatest effect on extraction yield and IC50. An increase in both responses occurred proportionally to temperature rise. On the other hand, ratio proved to be the most important factor for total chlorophylls, selected and total carotenoids. Ratio increase presented an inversely proportional effect on pigment composition.

#### 2.3.2. Reliability Analysis

**Figure 5:**
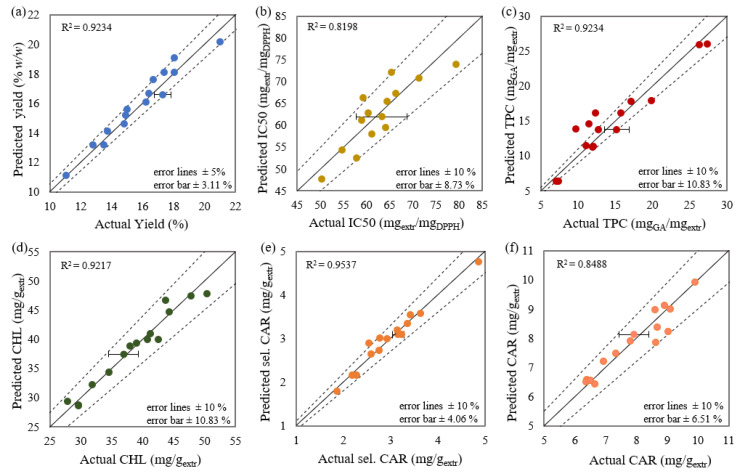
Experimental versus predicted values of (**a**) yield, (**b**) antioxidant activity, (**c**) total phenolic content, (**d**) total chlorophyll content, (**e**) selected carotenoid content, and (**f**) total carotenoid content. Error bars express experimental standard deviation.

The proposed models were considered acceptable according to the evaluation of the reliability tests and model adequacy measures presented in Table A1 (see Appendix A). In general, the examined responses presented satisfactory correlation proved by the high affinity of experimental and predicted values (Figure 5). However, both equations of antioxidant activity and phenolics were the least satisfactory regarding the noteworthy number of non-significant terms that were necessarily added for the hierarchical integrity maintenance in combination with the lower values of R^2^. Therefore, models of antioxidant activity and total phenolic content were considered useful tools for estimation but doubtful for accurate prediction.

#### 2.3.3. Synergistic Effect of the Independent Variables

**Figure 6:**
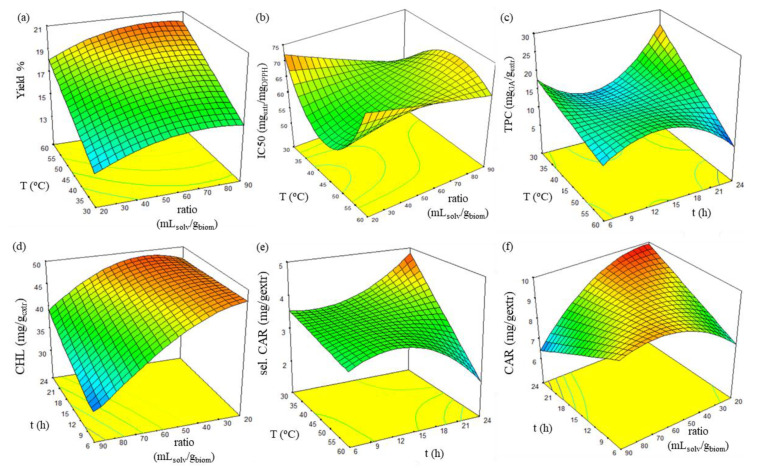
Response surface plot showing the combined effect of most significant variables for the (**a**) antioxidant activity, (**b**) total phenolic content, (**c**) total chlorophyll content, (**d**) selected carotenoid content, and (**e**) total carotenoid content. (**f**) The third absent independent variable refers to 30 °C, 24 h, and 20 mL_solv_/g_biom_ accordingly.

In order to understand the interactions and consequently determine the optimal extraction conditions and receive extracts with the best possible characteristics, surface plots of all responses were employed, as presented in Figure 6. Axial terms were selected based on the significance of their interaction term, as observed in Table A1 (see Appendix A).

Interaction of temperature and ratio significantly affected yield and antioxidant activity. According to Figure 6a, a single-curved surface describes the dependence of yield as a function of temperature and ratio for 24 h extraction. Individual ratio increase presents little effect on yield. However, a combined increase in ratio and temperature increases extraction yield significantly, and the maximum value is estimated at 60 °C and 90 mL_solv_/g_biom_.

Regarding antioxidant activity and according to Figure 6b, for 24 h extractions and ratio values above 50 mL_solv_/g_biom_, the initially improved antioxidant activity at 30 °C worsens with increasing temperature until 40 °C and then increases again until 60 °C. However, a reversal is observed for ratio values under 50 mL/gbiom with the presence of maximum antioxidant activity at around 42 °C and 20 mL_solv_/g_biom_ ratio.

Furthermore, a noteworthy synergistic effect of extraction temperature and duration on total phenolic and selected carotenoid content was observed. Corresponding response surface plots, shown in Figure 6c,e, illustrate this combined effect for a solvent-to-biomass ratio of 20 mL_solv_/g_biom_. Both responses present a similar complex locus. Local maximum values are observed at high temperatures for 13 h extractions. Temperature reduction down to 40–45 °C leaves phenolics and selected carotenoids intact to a relatively mediocre value regardless of extraction time, while further reduction in temperature leads to reversal of the initially observed phenomenon for total phenolic content and continuous reduction in selected carotenoid content with duration decrease. Recovery of maximum phenolic and selected carotenoid content is estimated at 30 °C and 24 h.

In addition, the important synergistic effect of temperature and ratio on chlorophyll content was confirmed. According to the curved surface of Figure 6d, extract deficient in chlorophylls is received under the lowest extraction duration and the highest ratio at 30 °C. An increase in duration leads to a slight improvement, but when combined with the ratio’s decrease, it significantly improves chlorophyll content.

Finally, evaluating the combination of duration and ratio for extraction at 30 °C (Figure 6f) reveals that maximum carotenoid content is estimated at the highest extraction duration and lowest ratio. Each individual duration decrease and ratio increase from optimum conditions are estimated to lead to a severe carotenoid reduction, while simultaneous parameter change is responsible for a slighter decrease.

#### 2.3.4. Extraction Process Optimization and Validation Experiment

The final goal of the present work was the determination of the simultaneously optimal extraction conditions of *C. vulgaris*. Since natural carotenoid prices (350–7500 USD/kg) are significantly higher than the ones of synthetic carotenoids (250–2000 USD/kg) [55], greater emphasis in determining the optimum conditions was given to selected and total carotenoid maximization at the expense of the rest responses. According to the optimization analysis (see Appendix A), the determined optimal conditions were 30 °C, 24 h, and 37 mL_solv_/g_biom_ for temperature, duration, and the solvent-to-biomass ratio of the extraction, respectively.

A confirmation experiment was carried out under the above conditions, and the results are presented in Table 3. Experimental extraction yield, chlorophyll content, selected and total carotenoids deviated from the corresponding predicted values less than 5%, indicating the satisfactory description of the requested responses and sufficient precision of models [56,57]. Deviation of antioxidant activity and total phenolics, on the other hand, was estimated at approximately 13 and 17%, respectively. These deviations could be justified due to the lower R^2^ of the corresponding models.

## 3. Materials and Methods

### 3.1. Materials

Commercial *C. vulgaris* biomass was provided in powder form by Go Superfoods Ltd. (Sheffield, UK). *C. vulgaris* was cultivated in natural water open ponds in South China, harvested with mesh screens, pretreated through milling, and spray dried. All referred stages are in accordance with strict regulations for human consumption-intended products. Chloroform, ethyl acetate, phenol crystals, orthophosphoric acid (analytical grade reagents), methanol (≥99.8%), tert-butyl-methyl ether (MTBE), water (HPLC grade reagents), and anhydrous sodium carbonate (99.5%) were purchased from Fisher Scientific International Inc. (Pittsburgh, PA, USA). Standard compounds of astaxanthin (≥98%), lutein (≥92%), and β-carotene (≥95%) for HPLC analysis were purchased from Acros Organics BVBA (Antwerp, Belgium), Extrasynthese SAS (Lyon, France), and Sigma Aldrich Co. (Saint Louis, MO, USA), respectively. Anhydrous D(+)-glucose (≥99.8%) and gallic acid (98%) (ACS reagents) were purchased from Acros Organics BVBA (Antwerp, Belgium). Analytical grade potassium chloride was purchased from Panreac Quimica SA (Barcelona, Spain), while 2,2-diphenyl-1-picrylhydrazyl (DPPH) free radical and Folin–Ciocalteu reagent were purchased from Sigma Aldrich Co. (Saint Louis, MO, USA) and Carlo Erba Reagents SAS (Milan, Italy), respectively.

### 3.2. Instrumentation

All devices used in this study are listed below. Drying processes were performed using a Gallenkamp OVA031.XX1.5 vacuum oven (A. Gallenkamp & Co., Ltd., London, UK) and combustion processes using a Thermolyne 47,900 furnace (Barnstead Thermolyne Corp., Ramsey, MN, USA). Ultrasound processes were carried out at an ambient temperature in an Elma D-7700 Transsonic Digital ultrasonic bath (Elma Schmidbauer GmbH, Singen, Germany) working at 35 kHz. Moreover, centrifugation, depending on sample volumes, was performed using an Eppendorf 5452 Mini Spin centrifuge (Eppendorf AG, Hamburg, Germany) and a Hermle centrifuge Z206-A (Hermle AG, Baden-Württemberg, Germany), while required filtrations were performed using ChromPure PTFE/L 0.45 μm filters (Membrane solutions, LLC, North Blend, OH, USA). Agitation during extraction processes was performed via a Carousel tech stirring hotplate (Radleys, Essex, UK), while intense sample stirring was performed using a vortex mixer Vortex-Genie^®^ 2 (Scientific industries Inc., Bohemia, NY, USA). Vacuum evaporation was performed using a Hei-VAP Advantage ML rotary evaporator (Heidolph Instruments GmbH & Co. KG, Bayern, Germany). Required spectrophotometric measurements were performed in a Shimadzu UV-1900i UV–Vis Spectrophotometer (Shimadzu Corporation, Kyoto, Japan) using 1 cm length quartz cuvettes. Determination of nitrogen was performed using a Speed Digester K-425 connected to a Scrubber K-415 for exhaust gas collection and a Kjelflex K-360 distillation device (Buchi Labortechnik AG, Flawil, Switzerland). Finally, high-performance liquid chromatography was performed using an HPLC device consisting of a Jasco LG-1580-04 gradient unit and a Jasco PU-1580 HPLC pump (Jasco Inc., Easton, MD, USA), a Rheodyne 7125 injector (Rheodyne Europe GmbH, Bensheim, Germany) with 20 μL loop, a Jones 7955 column chromatography heater (Jones Chromatography Limited, Wales, UK), and a Shimadzu SDP-M20A Diode Array Detector (DAD) (Shimadzu Corporation, Kyoto, Japan). In this study, the stationary phase was immobilized in a YMC C30 reversed-phase column, 5 μm, 250 × 4.6 mm I.D. (YMC Co., Ltd., Kyoto, Japan).

### 3.3. Methods

#### 3.3.1. Biomass Characterization

The chemical composition, as well as the moisture and ash content of the microalgal *C. vulgaris* biomass, were determined by applying relevant methods in triplicates as described below. Results are presented as AVG ± SD%, where AVG and SD stand for the average value and standard deviation of each triplicate.

##### Moisture Content

The content of moisture was determined through the loss-on-drying method. Samples of biomass, 0.1–0.2 g each, were vacuum dried at 100 mbar and 40 °C until weight stabilization, which typically occurred in less than 12 h. Each sample’s moisture was expressed as a percentage of dry to wet biomass (% *w*/*w*).

##### Ash Content

Combustion was performed for ash content determination. Samples of biomass, 0.2–0.5 g each, were placed into dried porcelain crucible and combusted at 550 °C until weight stabilization, which typically occurred in less than 3 h. The ash content, which indicates minerals of microalgae biomass, was expressed as a percentage of the amount of ash to dry biomass (% dw).

##### Lipid Content

Lipids were determined through the Folch method [58] as adapted by Araujo et al. [59], with additional modifications including reduced biomass samples and corresponding volumes of methanol and chloroform. Briefly, 0.1 g of biomass was homogenized with 0.5 mL methanol and sonicated for 3 min. Chloroform addition, 1 mL, followed, and the mixture was subjected to ultrasonic energy for 27 min. Constant temperature measurement and cool deionized water addition when bath temperature exceeded 28 °C contributed to ambient temperature levels maintenance during sonication. Regarding lipid separation, the mixture was centrifuged for 8 min at 13.400 rpm prior to filtration and in addition, the extracted biomass was exhaustively washed (3 times) with 1.5 mL of methanol/chloroform mixture (0.5 mL/1 mL) by vortexing for 5 s, centrifuging the mixture and filtering the supernatants. For all required filtrations, the same filter was used and finally washed with 1.5 mL of the same methanol/chloroform mixture. All supernatants (7.5 mL) were collected, mixed with 7.5 mL of potassium chloride (0.88% *w*/*v*) for removal of non-lipid impurities [60], and let to settle in a separating funnel for 5 h until the aqueous/higher phase and the oil/lower phase were separated by a distinct separating surface. After separation occurred, the oil phase, representing the lipid fraction, was collected with a simple outflow of the funnel and weighed in a pre-weighed flask after vacuum evaporation at 45 °C and 100 mbar. Finally, total lipid content was expressed as a percentage of the number of lipids to dry biomass (% dw).

##### Carbohydrate Content

The content of biomass in carbohydrates was determined according to the phenol-sulfuric method as described by Moheimani et al. [61]. Approximately 1000 μg of biomass were subjected to analysis and all absorbance measurements were performed at 485 nm. The concentration of carbohydrates was expressed in glycose equivalents and carbohydrate content as a percentage of the number of carbohydrates to dry biomass (% dw).

##### Protein Content

The protein content was determined through the Kjeldahl method [62]. Approximately 0.1 g of biomass samples were dried at 45 °C for 48 h prior to further analysis. The Kjeldahl method was performed according to the protocol provided by Büchi Labortechnik AG (Flawil, Switzerland) [63]. A slight modification occurred by replacing the commercial catalyst formulation with 3.72 g of K_2_SO_4_-CuSO_4_*5H_2_O-TiO_2_ (100:3:3 *w*/*w*/*w*). Moreover, the produced solution from the distillation step was manually titrated in the presence of bromocresol green/methyl red indicator, until the light blue color turned into light pink. Finally, total Kjeldahl nitrogen was multiplied with a nitrogen-to-protein factor of 4.78, a value suitable for microalgal biomass [64], and protein content was expressed as a percentage of the amount of protein to dry biomass (% dw).

#### 3.3.2. Conventional Extraction

##### Method Description

Conventional extraction was performed with about 1 g of *C. vulgaris* biomass. Sample and ethanol 90% *v*/*v* were loaded into a jacketed vessel, stirred at 500 rpm, and heated in the dark. A condenser was connected to the top of the vessel for the minimization of solvent losses. After extraction, the mixture was centrifuged for 8 min at 3.000 rpm. The supernatant was filtered and vacuum evaporated at 45 °C and 100 mbar. Dry microalgal extracts obtained after evaporation were stored at −18 °C until further analysis.

##### Experimental Design, Model Fitting, and Optimization

An FC-CCD method was used in the present study. The effect of three independent variables—namely, extraction temperature (T), extraction duration (t), and solvent-to-biomass ratio, was studied on six different responses—namely, yield, antioxidant activity (IC50), total phenolic content (TPC), chlorophylls (CHL), selected carotenoids (sel. CAR)—astaxanthin, lutein, and β-carotene—and total carotenoids (CAR). According to the FC-CCD, the independent variables were examined in three different levels (low, midpoint, high), resulting in three groups of design points. Firstly, the factorial points were consisted of all the possible combinations of low and high levels. Secondly, for the axial points, one factor was set equal to a low or high level and the rest were set to the midpoint level; thirdly, all the levels of the center point were set to the midpoint, which was deliberately repeated four times for accurate determination of the experimental error. The three levels of each variable were encoded as +1, 0, +1 for the high, midpoint, and low level, respectively, as shown in Table 4. All setpoint combinations of the experimental design are presented in Table 5.

Assessment of experimental data was performed through analysis of variance (ANOVA) using an equation in the following form:

Y = b_0_ + b_1_ × X_1_ + b_2_ × X_2_ + b_3_ × X_3_ + b_4_ × X_1_ × X_2_ + b_5_ × X_1_ × X_3_ + b_6_ × X_2_ × X_3_ + b_7_ × X_1_^2^ + b_8_ × X_2_^2^ + b_9_ × X_3_^2^ + b_10_ × X_1_ × X_2_ × X_3_ + b_11_ × X_1_^2^ × X_2_ + b_12_ × X_1_^2^ × X_3_ + b_13_ × X_1_ × X_2_^2^ + b_14_ × X_1_ × X_3_^2^ + b_15_ × X_2_^2^ × X_3_ + b_16_ × X_2_ × X_3_^2^ (9)

where Y stands for the examined response, b_0_–b_16_ stands for the coefficients, and X_1_–X_3_ stands for the chosen independent variables.

Where deemed necessary, transformation (e.g., natural log), Y′, of the initial response, Y, was applied and evaluated in a similar way, as described by Equation (10).

Y′ = f(Y) → Y = f(Y)(10)

However, determination of the significance of each factor and their interactions led to discarding insignificant terms, while maintaining the model’s hierarchy and ANOVA was repeated for the reduced model. The significance of each effect was determined through the Fisher’s statistical test (F-test) with a 95% significance level. Experimental design, modeling, and statistical analysis of the results were performed using the Design Expert^®^ Version 13 software trial (Stat-Ease Inc., Minneapolis, MN, USA).

#### 3.3.3. Extract Analysis

##### Antioxidant Activity

The antioxidant activity of *C. vulgaris* extracts was determined through the DPPH free radical scavenging assay, as described by Laina et al. [65]. Extracts were dissolved in methanol (40 mg/mL), and all absorbance measurements required were performed at 515 nm. Antioxidant activity was expressed in terms of half-maximal inhibitory concentration (IC50), with measurement units equal to mg of extract per mg of DPPH. Therefore, the lower the IC50 value, the higher the exhibited antioxidant activity of the extract. Protocol and calculation steps are provided in detail in Appendix B.1.

##### Total Phenolic Content

The total phenolic content was determined through the Folin–Ciocalteu assay, based on the modified method, in terms of volume reduction and thermal reaction acceleration, described by Drosou et al. [66]. Extracts were dissolved in methanol (10 g/L), and all measurements required were performed at 765 nm. Total phenolic content (TPC) was estimated as the mass ratio of gallic acid equivalent to extract (mg_GA_/g_extr_).

##### Total Chlorophylls and Carotenoids

Equations provided by Jeffrey et al. were used for chlorophyll (a, b, and c) [67] and total carotenoid content [68] determination of the extracts, using 90% acetone as a solvent. All absorbance measurements required were performed at the wavelengths of 480 and 510 nm for carotenoids and 630, 647, and 664 nm for chlorophylls. The sums of individually determined chlorophyll a, b, and c led to the estimation of total chlorophylls, while total chlorophyll (CHL) and carotenoid content (CAR) were expressed in mass ratios of the corresponding compound to extract (mg/g_extr_). The equations used are provided in Appendix B.3.

##### Selected Carotenoids of Astaxanthin, Lutein, and β-Carotene

The received extracts were also subjected to reversed-phase–high-performance liquid chromatography (RP–HPLC) for carotenoid analysis. The mobile phase consisted of methanol, methyl tert-butyl ether (MTBE), and aq. phosphoric acid 1% *v*/*v*. Separation was achieved with a linear gradient, presented in Table 6, within 35 min, adjusted by Fuji Chemical Industry Co., Ltd. (Toyoma, Japan), as reported by Stramarkou et al. [69], at a column temperature of 35 °C and flow rate of 1 mL/min.

The selected carotenoids of astaxanthin, lutein, and β-carotene were identified by comparison of retention times, and absorbance spectra of external standards (Appendix B.2) and quantified through corresponding standard reference curves. Both external standards and *C. vulgaris* extracts were dissolved in ethyl acetate, with concentrations varying from 1 to 25 mg/L and from 5 to 10 mg/mL, respectively. The sum of individually determined astaxanthin, lutein, and β-carotene led to the selected carotenoid content (sel. CAR) estimation, which was expressed in a mass ratio of compound to extract (mg/g_extr_).

## 4. Conclusions

The present study determined the effects of extraction temperature, duration, and solvent-to-biomass ratio, on the simultaneous recovery of several bioactive compounds from *C. vulgaris* biomass through conventional extraction with aq. ethanol 90% *v*/*v*. An RSM of FC-CCD was employed for the experimental design, the response study of extraction yield, extract’s antioxidant activity, total phenolic, chlorophyll, carotenoid content and selected carotenoid content of astaxanthin, lutein, and β-carotene, as well as the extraction process optimization.

Temperature variation proved to be the most important factor for extraction yield and antioxidant activity, while ratio variation presented the most important effect on pigment composition. Regarding the factors’ synergistic effect, the combined factor of temperature and ratio was responsible for the most significant impact on yield, while the combination of duration and ratio highly affected antioxidant activity and chlorophyll content. Moreover, the combination of temperature and duration presented a significant effect on phenolic and selected carotenoid content, while the synergistic effect of all three factors highly affected total carotenoids.

Developed models’ verification confirmed the capability of response prediction under acceptable confidence levels and allowed reliable process optimization slightly oriented to carotenoid recovery. Thus, the optimal extraction conditions obtained were 30 °C for 24 h with 37 mL_solv_/g_biom_ solvent-to-biomass ratio. Experiment under optimal conditions was performed for models’ verification, leading to 15.39% *w*/*w* yield, 52.58 mg_extr_/mg_DPPH_ antioxidant activity (IC50), total phenolic, chlorophyll, and carotenoid content of 18.23, 53.47, and 9.92 mg/g_extr_, respectively, and 4.12 mg/g_extr_ selected carotenoid content.

Such a comprehensive study provides useful information for comparison with corresponding studies of other biomass sources, as well as other conventional or innovative extraction methods. Finally, the provided information might be valuable for process simulation and scale-up purposes.

## Figures and Tables

**Table 1:** Primary composition of the commercial *Chlorella vulgaris* biomass.

Primary Composition	% ^1^
Lipid	22.17 ± 0.46
Carbohydrate	33.84 ± 1.33
Protein	44.48 ± 0.77
Ash	5.63 ± 0.06
Moisture	2.32 ± 0.12

^1^ All values except moisture are expressed on dry basis (dw).

**Table 2:** Three-factor FC-CCD with the corresponding responses of yield, antioxidant activity (IC50), total phenolic content (TPC), chlorophyll (CHL), selected carotenoid (sel. CAR)—astaxanthin, lutein, and β-carotene—and total carotenoid (CAR) content.

Run	T (°C)	t (h)	Ratio (mL_solv_/g_biom_)	Yield (% *w*/*w*)	IC50 (mg_extr_/mg_DPPH_)	TPC (mg_GA_/g_extr_)	CHL (mg/g_extr_)	sel. CAR (mg/g_extr_)	CAR (mg/g_extr_)
1	30	15	55	13.75	57.77	11.14	44.34	2.93	8.92
2	30	6	20	11.06	50.31	17.15	50.43	3.63	7.35
3	30	6	90	13.49	34.73	19.89	27.86	2.28	9.11
4	30	24	20	12.82	65.29	26.28	47.82	4.87	9.90
5	30	24	90	14.91	61.11	27.35	39.00	3.36	6.65
6	45	6	55	15.02	60.31	11.49	41.23	2.54	8.67
7	45	15	20	14.82	54.72	12.80	40.76	3.42	8.63
8	45	15	55	16.55	55.85	17.79	40.17	3.04	8.61
9	45	15	55	17.02	70.78	15.22	37.06	3.18	7.84
10	45	15	55	17.84	60.88	14.50	36.84	3.06	8.07
11	45	15	55	17.77	65.47	13.29	33.45	3.36	7.18
12	45	15	90	16.40	71.29	9.72	29.69	2.59	6.40
13	45	24	55	16.70	58.86	12.37	34.53	2.78	7.80
14	60	6	20	16.24	66.25	12.02	43.74	3.23	8.61
15	60	6	90	17.40	64.03	12.11	29.67	2.76	6.36
16	60	24	20	18.08	79.41	7.46	38.01	2.18	6.93
17	60	24	90	21.01	64.42	7.07	31.85	1.88	6.52
18	60	15	55	18.04	59.28	15.80	42.59	3.14	9.05

**Table 3:** Optimal conditions for the conventional extraction of bioactive compounds from *C. vulgaris*.

T (°C)	t (h)	Ratio (mL_solv_/g_biom_)
30	24	37
**Response**	**Predicted**	**Actual**
Yield (% *w*/*w*)	14.36	15.39
IC50 (mg_extr_/mg_DPPH_)	68.40	52.58
TPC (mg_GA_/g_extr_)	25.86	18.23
CHL (mg/g_extr_)	48.83	53.47
sel. CAR (mg/g_extr_)	4.42	4.12
CAR (mg/g_extr_)	9.75	9.92

**Table 4:** The levels of the rotatable central composite design of *C. vulgaris* extraction.

RSM/Face-Centered Central Composite Design (Alpha = 1)					
Variable	Factor	Units	−1 Level	0 Level	+1 Level
T	X_1_	°C	30	45	60
t	X_2_	h	6	15	24
Ratio	X_3_	mL_solv_/g_biom_	20	55	90

**Table 5:** Set of the experimental design of *C. vulgaris* maceration formed by RSM–FC-CCD.

RSM/Face-Centered Central Composite Design (Alpha = 1)							
		Coded Factors			Real Variables		
Run	Type	X_1_	X_2_	X_3_	T (°C)	T (h)	Ratio (mL_solv_/g_biom_)
1	Axial	−1	0	0	30	15	55
2	Factorial	−1	−1	−1	30	6	20
3	Factorial	−1	−1	1	30	6	90
4	Factorial	−1	1	−1	30	24	20
5	Factorial	−1	1	1	30	24	90
6	Axial	0	−1	0	45	6	55
7	Axial	0	0	−1	45	15	20
8	Central	0	0	0	45	15	55
9	Central	0	0	0	45	15	55
10	Central	0	0	0	45	15	55
11	Central	0	0	0	45	15	55
12	Axial	0	0	1	45	15	90
13	Axial	0	1	0	45	24	55
14	Factorial	1	−1	−1	60	6	20
15	Factorial	1	−1	1	60	6	90
16	Factorial	1	1	−1	60	24	20
17	Factorial	1	1	1	60	24	90
18	Axial	1	0	0	60	15	55

**Table 6:** The linear gradient of the mobile phase applied in RP-HPLC.

Time (Min)	Methanol (% *v*/*v*)	MTBE (% *v*/*v*)	aq. Phosphoric Acid_1%*v*/*v*_ (% *v*/*v*)
0	81	15	4
15	66	30	4
23	16	80	4
27	16	80	4
27.1	81	15	4
35	81	15	4

**Figure A1:**
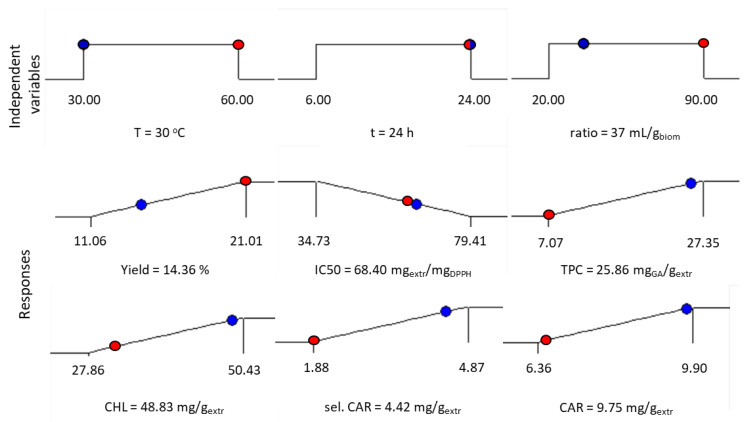
Illustration of the overall optimal (blue dots) and optimal yield (red dots) conditions and the corresponding responses in the form of ramps. Predicted values of overall optimal independent variables and responses are also included.

**Figure A2:**
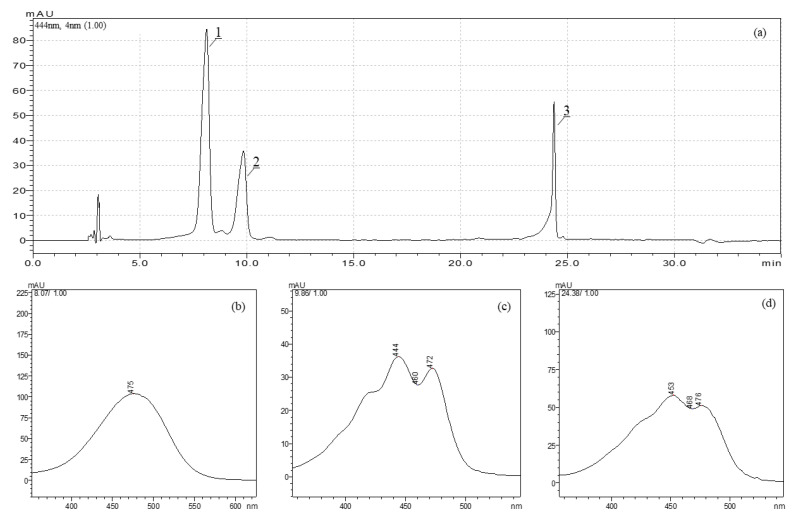
(**a**) Chromatogram of (1) astaxanthin, (2) lutein, and (3) β-carotene external standard mixture at 444 nm and absorbance spectra of (**b**) astaxanthin, (**c**) lutein, and (**d**) β-carotene.

## Data Availability

Additional data for this study are not available on a public database; the corresponding author can provide them upon request.

## Sample Availability

Samples of the compounds are not available from the authors.

## Appendix A. Evaluation of the ANOVA Results and Optimization

In the ANOVA, the F and *p*-values of the model, individual equation term, and lack of fit determined their statistical significance. More specifically, a high F-value and low *p*-value (<0.05) characterize the corresponding term as significant, in contrast to a lower F-value and higher *p*-value (>0.1), which refer to insignificant terms. Additionally, the coefficient of determination, R^2^, is useful for the model’s evaluation. A high R^2^ value is desirable and shows how close experimental and predicted values are. Finally, adequate precision, Adeq Prec, provides information about the model’s accuracy. Values of Adeq Prec exceeding 4 prove the model’s sufficiency.

Table A1 presents F- and *p*-values of the examined models, significant equation terms and lack of fit, as well as adequacy measures of R^2^ and Adeq Prec. High F-values and low *p*-values of all the described models described through Equations (1), (2), (4), (5), (7) and (8) indicate their significance and reliability, while low F-value and high *p*-value of lack of fits show that they are insignificant and prove models’ appropriateness and adequacy of data. In addition to extraction yield, cubic terms were included in the model equations of all other responses. The low *p*-value of these third-degree factors proved their significance and essential presence for satisfactory response description and prediction. All responses presented considerably high R^2^. The regression model of selected carotenoids appeared to have the highest R^2^ (0.9537), followed by the models of total chlorophylls (0.9248), yield (0.9234), total phenolics (0.8658), total carotenoids (0.8488), and total antioxidant activity (0.8198). Finally, high adequate precision of all responses (Adeq Prec > 4) proved models’ accuracy.

**Table A1:** Main ANOVA results of the examined responses derived from the FC-CCD RSM and model adequacy measures.

**Yield**				**Antioxidant Activity**			
Source		F-Value	*p*-Value	Source		F-Value	*p*-Value
Yield Model		39.19	<0.0001	IC50 Model		5.12	0.0124
T		111.13	<0.0001	T		13.08	0.0056
t		19.29	0.0007	T^2^*ratio		7.32	0.0242
ratio		18.91	0.0008	Lack of Fit		1.01	0.5418
ratio^2^		7.45	0.0172	R^2^	0.8198	Adeq Prec	9.130
Lack of Fit		1.56	0.3937				
R^2^	0.9234	Adeq Prec	23.14				
**Total Phenolic Content**				**Total Chlorophyll Content**			
Source		F-value	*p*-value	Source		F-value	*p*-value
TPC Model		11.83	0.0003	CHL Model		13.84	0.0003
T*t		13.46	0.0037	T		7.57	0.0224
T*t^2^		19.63	0.0010	ratio		70.19	<0.0001
Lack of Fit		2.04	0.3008	t*ratio		11.24	0.0085
R^2^	0.8658	Adeq Prec	12.49	T^2^		10.84	0.0093
				ratio^2^		8.36	0.0178
				T^2^*t		5.31	0.0467
				Lack of Fit		0.57	0.7421
				R^2^	0.9248	Adeq Prec	11.47
**sel. Carotenoid Content**				**Total Carotenoid Content**			
Source		F-value	*p*-value	Source		F-value	*p*-value
sel.CAR Model		29.39	<0.0001	CAR Model		4.99	0.0168
ratio		58.62	<0.0001	T		5.32	0.0500
T*t		65.86	<0.0001	ratio		10.81	0.0111
T*ratio		15.86	0.0026	ratio^2^		7.05	0.0291
T*t^2^		17.73	0.0018	T*t*ratio		15.63	0.0042
Lack of Fit		1.80	0.3392	Lack of Fit		1.09	0.5041
R^2^	0.9537	Adeq Prec	24.17	R^2^	0.8488	Adeq Prec	7.64

Design Expert^®^ 13 software was used to define the optimal extraction conditions and predict the values of each response. The independent variables were chosen to range in their domain without any restriction. All the examined responses were set to maximize, while IC50 was set to minimize. A weight factor of 2.5 was applied on the responses of selected and total carotenoid content, whereas all other responses maintained a neutral weight factor equal to 1. The choice of the referred carotenoid weight factor emerged after several attempts that would reach a satisfying theoretical carotenoid recovery without significant deterioration of the other responses. Optimization results, both optimal conditions and response prediction, are illustrated in Figure A1. According to the performed analysis, the determined optimal conditions were 30 °C, 24 h, and 37 mL_solv_/g_biom_ for temperature, duration, and the solvent-to-biomass ratio of the extraction, respectively. Comparing the theoretical overall optimal conditions and responses that emerged during optimization (Figure A1, blue dots) with the optimal yield run (Figure A1, red dots) revealed that the former one, with lower energy and solvent demands, was estimated to present a reduced yield by 30%, a similar antioxidant activity but nevertheless superior bioactive compound characterization. Slightly reduced extraction yield was overshadowed by the remarkably higher extract quality, and thus, the appropriate impact factor used was justified.

## Appendix B. Supplementary Data of Extract Analysis Protocols

### Appendix B.1. Antioxidant Activity

DPPH solution in methanol (0.03 mg/mL) was prepared in a dark glass container and stirred for 15 min at room temperature before use. DPPH solution (3.9 mL) and a sample of methanolic extract solution (0.1 m) of different concentrations were mixed and incubated for 30 min in a dark place at ambient temperature, and then, absorbance was measured at 515 nm. Plain DPPH solution served as control, while a mixture of methanol (3.9 mL) and extract sample (0.1 mL) served as blank. Radical scavenging (SCA) was expressed as a percentage (%) and calculated using the following equation:

SCA = 100 × [1 − (A_sample_ − A_blank_)/A_control_] (A1)

where A_sample_, A_blank_, and A_control_ stand for the absorbance of the sample, blank and control, respectively.

Different sample concentrations were used to acquire a linear scavenging curve, with x axes indicating sample concentration (mg/mL), and y axes indicating SCA (%), and subsequently, extract concentration responsible for 50% inhibition of DPPH (mg_extr_/mL) was obtained by setting SCA, equal to 50%. Finally, half-maximal inhibitory concentration (IC50), with measurement units equal to mg of extract per mg of DPPH, expressed antioxidant activity using the following equation:

IC50 = (V_sample_ × C_50%_)/(0.5 × V_DPPH_ × C_DPPH_) (A2)

where V_sample_ and V_DPPH_ stand for the volume of sample and DPPH used (0.1 and 3.9 mL, respectively), C_DPPH_ stands for the initial concentration of untreated DPPH (mg_DPPH_/mL), while C_50%_ stands for the sample concentration (mg_extr_/mL) responsible for 50% neutralization of DPPH as emerged from the scavenging curve.

### Appendix B.2. Detection of Selected Carotenoids Using RP–HPLC

According to Figure A2a, astaxanthin was first detected (peak 1) followed by lutein (peak 2), and finally, β-carotene (peak 3). Figure A2b–d reveals the determination of wavelength detection, which was achieved at 444 nm for lutein, 453 nm for β-carotene, and 475 nm for astaxanthin.

### Appendix B.3. Total Chlorophylls and Carotenoids

The equations used for chlorophyll and carotenoid determination, according to Jeffrey and Humphrey [67] are presented as follows:

c_a_ = 11.85 ∗ A_664_ − 1.54 ∗ A_647_ − 0.08 ∗ A_630_ (A3)

c_b_ = 21.03 ∗ A_647_ − 5.43 ∗ A_664_ − 2.66 ∗ A_630_ (A4)

c_c_ = 24.52 ∗ A_630_ − 1.67 ∗ A_664_ − 7.60 ∗ A_647_ (A5)

c_chlor._ = c_a_ + c_b_ + c_c_ (A6)

c_carot._ = 7.60 ∗ A_480_ − 1.49 ∗ A_510_ (A7)

where A_664_, A_647_, A_630_, A_480_, A_510_ stand for the absorbance measurement at 664, 647, 480, and 510 nm, c_a_, c_b_, c_c_, c_chlor._, and c_carot._ stand for the concentration of chlorophyll a, b, c, total chlorophylls, and total carotenoids, respectively (μg/mL).

Total chlorophyll (C_chlor._) and carotenoid (C_carot._) content was expressed in mass units (mg/g_extr_) using the following equation:

C_i_ = 10^3^ ∗ c_i_/C_sample_, i = chlor., carot. (A8)

where C_sample_ stands for the sample concentration (g_extr_/mL).
